# The expanding role of IAP antagonists for the treatment of head and neck cancer

**DOI:** 10.1002/cam4.6011

**Published:** 2023-05-02

**Authors:** Vikash Kansal, Brendan L. C. Kinney, Srijayaprakash Uppada, Nabil F. Saba, William A. Stokes, Zachary S. Buchwald, Nicole C. Schmitt

**Affiliations:** ^1^ Department of Otolaryngology – Head and Neck Surgery Emory University Atlanta Georgia USA; ^2^ Winship Cancer Institute Emory University Atlanta Georgia USA; ^3^ Department of Radiation Oncology Emory University Atlanta Georgia USA; ^4^ Department of Hematology and Medical Oncology Emory University Atlanta Georgia USA

**Keywords:** birinapant, head and neck cancer, IAP antagonist, SMAC mimetic, squamous cell carcinoma, tolinapant, xevinapant

## Abstract

Inhibitors of apoptosis proteins (IAPs) inhibit the intrinsic and extrinsic cell death pathways, promoting cell survival. Antagonists of these pathways are under study as anti‐cancer therapeutics. A high proportion of head and neck squamous cell carcinomas (HNSCCs) have genomic alterations in IAP pathways, resulting in the dysregulation of cell death pathways and rendering them susceptible to IAP antagonist therapy. Preclinical studies suggest IAP antagonists, also known as second mitochondria‐derived activator of caspases mimetics, may be effective treatments for HNSCC, especially when combined with radiation. Mechanistic studies have shown both molecular mechanisms (i.e., enhanced cell death) and immune mechanisms (e.g., immunogenic cell death and T‐cell activation), underlying the efficacy of these drugs in preclinical models. Phase I/II clinical trials have shown promising results, portending a future where this class of targeted therapies becomes incorporated into the treatment paradigm for head and neck cancers.

IAP antagonists have shown great promise for head and neck cancer, especially in combination with radiation therapy. Here, we review recent preclinical and clinical studies on the use of these novel targeted agents for head and neck cancer.

## Graphical abstract

IAP Antagonists for Head and Neck Squamous Cell Carcinoma (HNSCC).

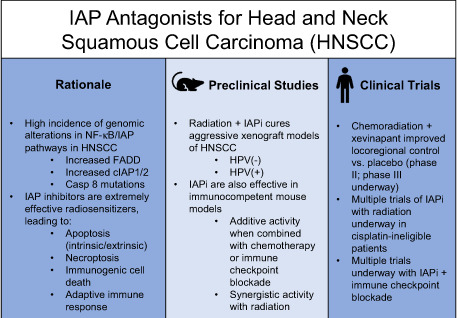

## 1 INTRODUCTION

Drugs antagonizing specific signaling pathways to induce cell death are under rigorous study for a variety of treatment‐refractory solid tumors. In cases where the signaling pathways in question are rare, targeted therapies offer an opportunity for precision medicine. However, the ideal targeted therapies will be effective for most patients with a given cancer, obviating the need for pretreatment molecular screening.

Head and neck squamous cell carcinoma (HNSCC) encompasses two distinct disease processes: oropharyngeal carcinoma driven by high‐risk human papillomavirus (HPV) and HPV‐negative HNSCC, which is typically linked to heavy use of tobacco and/or alcohol. Cetuximab, a chimeric mouse/human anti‐EGFR antibody, was the first FDA‐approved targeted agent for HNSCC. However, despite EGFR overexpression in 90% of head and neck cancers, cetuximab has shown inferior efficacy compared with standard cisplatin chemotherapy for HNSCC.^1^ As a result, there is still an unmet need for effective targeted therapies to treat HNSCC.

Small‐molecule drugs inhibiting the inhibitor of apoptosis proteins (IAPs) have shown promising activity in both HPV‐driven and HPV‐negative HNSCC, both in preclinical models and early clinical studies. IAP pathways are activated by radiation therapy (RT); thus IAP antagonists are highly effective as radiosensitizers. In this review, we introduce the IAP signaling pathways, highlight preclinical studies of IAP antagonists along with mechanistic insights, and summarize clinical trials incorporating IAP antagonists for head and neck cancer.

### 1.1 Inhibitor of apoptosis proteins and related pathways

**FIGURE 1:**
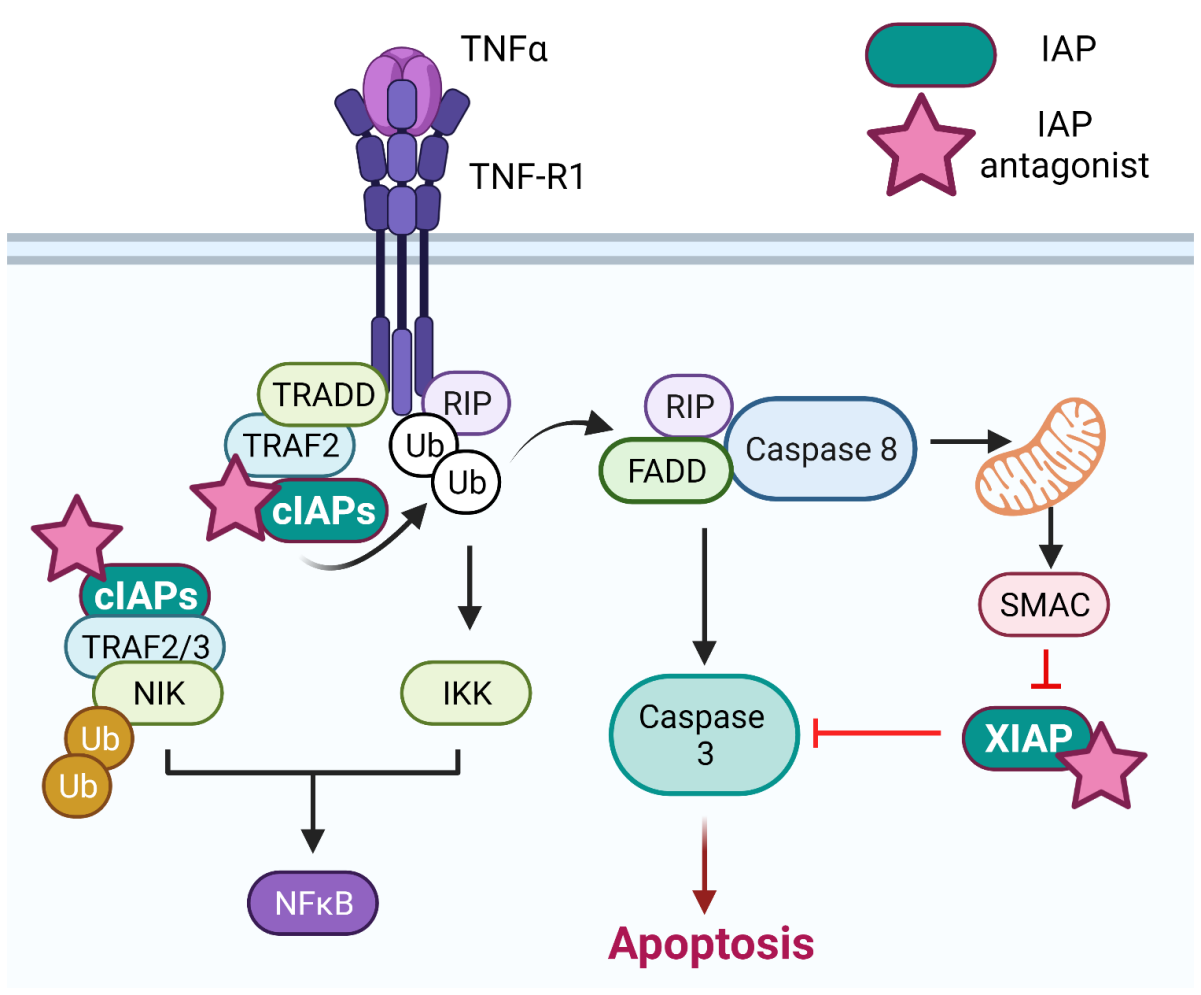
Schematic showing inhibitors of apoptosis protein (IAP) pathways and activity of IAP antagonists (pink stars). Created with Biorender.com.

IAPs are a family of proteins that inhibit apoptosis or programmed cell death and are often overexpressed in cancer cells, contributing to their resistance to chemotherapy and RT. IAP inhibitors (IAPi) are a group of small molecules that target IAPs and promote apoptosis in cancer cells.^2^, ^3^, ^4^, ^5^, ^6^, ^7^, ^8^ The detailed mechanism of IAPi involves the following steps^2^, ^3^, ^4^, ^5^, ^6^, ^7^, ^8^:

1. Binding of IAPi to IAP proteins: Binding of IAPi to IAP proteins (Figure 1) involves the interaction of the inhibitor molecule with the BIR (baculoviral IAP repeat) domains of IAPs. The BIR domains are regions of the IAP protein that are responsible for the interaction with other proteins involved in apoptosis, such as caspases.
2. Degradation of IAPs: The binding of IAPi to the BIR domains of IAPs induces a conformational change in the IAP protein, leading to the exposure of a degradation signal. The degradation signal serves as a recognition site for E3 ubiquitin ligases, which attach ubiquitin molecules to the IAP protein. The ubiquitinated IAP protein is thereby recognized and subsequently degraded by the proteasome.
3. Activation of caspases: IAPs inhibit caspases by binding them directly and inhibiting their activation. By removing IAPs, IAPi allow caspases to initiate the apoptotic cascade. Once activated, caspases cleave a variety of downstream targets, including the Bcl‐2 family of proteins, which regulate mitochondrial membrane permeability and release of apoptotic factors such as cytochrome c.
4. Induction of death receptor expression: Some IAPi can induce the expression of death receptors, such as tumor necrosis factor (TNF) receptor 1, which can activate caspases and trigger apoptosis. Death receptors are transmembrane proteins that bind to ligands such as TNF‐α and Fas‐L, leading to the activation of caspases via the extrinsic apoptotic pathway.

XIAP (X‐linked inhibitor of apoptosis) and cIAP1/2 (cellular inhibitor of apoptosis 1 and 2) are members of the IAP family. As described above, they are important regulators of apoptosis and play a critical role in maintaining the balance between cell survival and death. XIAP is the most potent inhibitor of caspases, which are the key enzymes that execute apoptosis. XIAP binds to and inhibits caspases 3, 7, and 9, thereby preventing the activation of the caspase cascade and the induction of apoptosis. XIAP also has other cellular functions, such as the regulation of NF‐κB (nuclear factor kappa‐light‐chain‐enhancer of activated B cells) signaling and the control of protein degradation.

cIAP1 and cIAP2 are closely related proteins that function as E3 ubiquitin ligases. They regulate NF‐κB signaling by promoting the ubiquitination and degradation of proteins involved in the NF‐κB pathway. cIAP1/2 also regulates the activity of caspases by binding to and inhibiting caspases 3, 7, and 9. Additionally, cIAP1/2 is involved in regulating the stability of the TNF receptor family members, which leads to the activation of the NF‐κB pathway.

SMAC (second mitochondria‐derived activator of caspases) is a protein that plays a crucial role in the regulation of apoptosis. SMAC is released from mitochondria into the cytosol during the induction of apoptosis, where it binds and inhibits IAPs. SMAC contains a conserved N‐terminal tetrapeptide sequence, AVPI (Ala‐Val‐Pro‐Ile), which is essential for its binding to IAP proteins. The AVPI sequence binds to the BIR3 (baculovirus IAP repeat 3) domain of IAP proteins, disrupting their ability to inhibit caspases. The binding of SMAC to IAP proteins releases the inhibition of caspases, which leads to the activation of the caspase cascade and the induction of apoptosis. In addition to its role in caspase activation, SMAC also plays a role in the regulation of NF‐κB (nuclear factor kappa‐light‐chain‐enhancer of activated B cells) signaling. SMAC can also bind and inhibit TRAF2 (TNF receptor‐associated factor 2), which is involved in the activation of the NF‐κB pathway.

The development of SMAC mimetics, small molecules that mimic the AVPI sequence of SMAC, has emerged as a promising strategy for the treatment of cancer. SMAC mimetics bind to the BIR3 domain of IAP proteins and release the inhibition of caspases, leading to the induction of apoptosis in cancer cells. SMAC mimetics also inhibit canonical NF‐κB signaling while promoting the alternative/noncanonical NF‐κB pathway, though this appears to be a more minor effect versus the effects on cell death signaling.^2^ SMAC mimetics have shown promising results in preclinical and clinical studies as a potential treatment for various types of cancer.

Apoptosis can occur via intrinsic or extrinsic pathways, and IAPi/SMAC mimetics can actually inhibit both pathways of cell death. The intrinsic pathway is activated by internal cellular stressors, including DNA damage induced by RT or chemotherapy, leading to the release of cytochrome c from the mitochondria into the cytosol. Cytochrome c then binds to Apaf‐1, which recruits and activates caspase‐9, leading to the activation of downstream caspases and ultimately apoptosis. IAPi can sensitize cells to the intrinsic pathway by preventing the inhibition of caspases by IAPs, allowing them to be activated and promoting apoptosis.^9^ The extrinsic pathway is activated by the binding of death ligands, such as Fas ligand or TNF‐α, to their respective receptors on the cell surface. This activates the recruitment and assembly of the death‐inducing signaling complex, which contains caspase‐8 or caspase‐10, leading to their activation and downstream caspase activation. IAPi can also sensitize cells to the extrinsic pathway by preventing the inhibition of caspases by IAPs.^10^ IAPi can promote both intrinsic and extrinsic pathways of apoptosis by preventing the inhibition of caspases by IAPs, leading to increased activation of converging downstream caspase pathways and ultimately cell death. Given that the intrinsic pathway is heavily induced by RT, blocking the extrinsic pathway as well is an appealing aspect of IAPi and likely a major component of their radiosensitizing properties. IAPi can also promote necroptotic cell death,^2^, ^11^, ^12^, ^13^ which may be superior to apoptotic tumor cell death in its ability to induce an immune response in the tumor microenvironment.^14^

In summary, IAPi/SMAC mimetics promote apoptosis in cancer cells by binding to and degrading IAPs, activating caspases, inducing death receptors, and blocking inhibitors of caspase activation. These mechanisms make IAPi a promising class of therapeutic agents for the treatment of cancer.

### 1.2 Rationale for IAP antagonists in HNSCC

The IAP family of proteins may serve as survival factors in a variety of cancer types. However, HNSCC, along with gynecologic malignancies, may be most susceptible based on a high incidence of genomic alterations in IAP pathways. NF‐κB signaling pathways are an important component of HNSCC pathogenesis.^2^ Eytan et al. queried the Cancer Genome Atlas and demonstrated that 60% of HNSCC cases (including HPV‐positive and ‐negative) had alterations in Fas‐associated death domain (*FADD*), baculoviral IAP repeat containing 2/3 (*BIRC2/3*, the genes encoding cIAP1/2), and/or caspase 8. High expression of FADD or cIAP1 is associated with advanced disease stage and poor survival outcomes,^15^, ^16^, ^17^ highlighting the need for escalated therapy in these patients. This may be particularly true in patients with Fanconi anemia (FA), a rare condition that incurs inherent susceptibility to the development of oral cancer. It was recently shown that oral cancer cells associated with FA exhibit marked overexpression of *BIRC2/BIRC3*, with inherent sensitivity to IAPi.^18^ FA patients are hypersensitive to the effects of standard chemoradiation; thus, IAPi may be particularly useful in FA‐driven oral cancers.^18^

### 1.3 Preclinical efficacy studies

Numerous IAPi have been tested for anti‐tumor activity in HNSCC cell lines. In some cell lines, SMAC mimetics by themselves induced cell death in the form of apoptosis or necroptosis.^19^, ^20^ However, in most cases, IAPi sensitized the cell lines to extrinsic TNF‐α or TNF‐related apoptosis‐inducing ligand (TRAIL), often with synergistic activity when both TNF/TRAIL and a SMAC mimetic were added.^15^, ^19^, ^20^, ^21^, ^22^, ^23^, ^24^, ^25^, ^26^ Several studies by multiple independent research groups have shown synergistic activity of SMAC mimetics when combined with radiation in mice inoculated with xenografts of human HNSCC cell lines, including HPV‐positive/negative models and four different IAPi (birinapant, xevinapant, tolinapant, and LCL161). The anti‐tumor activity of these combinations was remarkable in these aggressive xenograft models, resulting in the cure of most tumor‐bearing mice.^17^, ^20^, ^25^, ^27^ Blocking of TNF‐α with a depleting antibody reversed these effects, underscoring the critical role of TNF‐α in the enhancement of cell death by IAPi + RT.^20^ The fact that xenografts were used in immunodeficient animals for most of these studies suggests that massive cell death can be achieved with IAPi + RT alone, without an intact immune system; in this situation, the lack of T lymphocytes suggests that the increase in TNF may be coming from inflammatory, stromal, or tumor cells themselves. These points are important to keep in mind when rationally designing clinical trials combining new agents with standard chemoradiation, which has immunosuppressive effects.^28^, ^29^ Combinations of IAPi + cytotoxic chemotherapy have also been trialed in mouse models of HNSCC, but the additive effects were modest.^15^, ^30^

### 1.4 Molecular and immune mechanisms of IAP antagonism in HNSCC

As noted above, the production of TNF‐α and TRAIL renders tumor cells susceptible to IAPi. Thus, any therapy that increases TNF‐α production (e.g., RT or cytotoxic chemotherapy) may enhance sensitivity to IAPi. Another study focusing on caspase 8 revealed that inactivating caspase 8 mutations rendered HNSCCs particularly susceptible to birinapant + RT, and this phenomenon was dependent on tumor cells maintaining RIP3 expression.^19^

Although most murine in vivo studies with SMAC mimetics to date have used immunodeficient xenograft models, our group has previously explored the effects of IAPi + RT with and without immune checkpoint blockade in immunocompetent, synergistic models of HNSCC. In the mouse oral cancer 1 model, a majority of tumor‐bearing mice were cured when tolinapant was combined with RT and anti‐PD‐1 therapy.^30^ Functional and marker studies of tumors and tumor‐draining lymph nodes from mice treated with tolinapant + RT showed enhanced T‐cell activation including increased interferon‐γ production.^30^, ^31^ Ex vivo co‐culture experiments in HNSCC models have shown that tolinapant enhances T‐cell killing of tumor cells.^31^ Indeed, by enhancing noncanonical NF‐κΒ signaling and TNF‐α production, IAPi have been shown to enhance T‐cell costimulation, macrophage function, NK cell function, and numerous other aspects of innate and adaptive anti‐tumor immunity, which have been previously reviewed.^32^

Radiation is a known inducer of immunogenic cell death (ICD), a well‐described phenomenon that includes cell surface expression of calreticulin/heat shock proteins and secretion of HMGB1, ATP and type I interferon, ultimately leading to dendritic cell activation.^33^ The finding of enhanced dendritic cell activation with IAPi + RT versus RT alone suggested enhanced ICD by IAPi, which was confirmed in multiple syngeneic mouse models of HNSCC.^31^ Other tumor cell‐intrinsic mechanisms of IAPi, when combined with TNF‐α, include enhanced expression of MHC class I and other antigen processing machinery required for the presentation of tumor neoantigens to T cells.^31^

Data on the efficacy of IAPi in HPV‐positive versus HPV‐negative models are limited. One in vitro study showed only modest radiosensitizing effects of the IAPi LCL161 when used in HPV‐positive cell lines, versus robust activity in HPV‐negative cell lines.^17^ However, the cell lines in that study did not appear to overexpress FADD or cIAP1/2, though TCGA studies suggest these alterations are seen in a subset of HPV‐positive tumors.^27^ However, tolinapant, which inhibits cIAP1/2 and XIAP, did sensitize cell lines to TNF‐α, TRAIL, or radiation, in part by stabilizing p53 expression; inhibition of necroptosis had minimal effect, suggesting that tolinapant acts primarily via apoptotic pathways in HPV‐positive tumor cells.^20^ Synergistic activity was seen when RT was combined with tolinapant in a xenograft using the HPV+ UPCI‐SCC‐90 cell line.^20^ These results suggest that IAPi may be useful even in HPV‐positive tumors that lack genomic alterations in IAP pathways.

### 1.5 Clinical trials of IAP antagonism

Clinical trials with four different IAP antagonists have focused specifically on HNSCC (Tables 1 and 2). Common adverse effects reported with multiple SMAC mimetics include cytopenias, elevations in serum amylase/lipase, and (rarely) facial nerve weakness.^36^, ^37^, ^38^ A trial using tolinapant as monotherapy showed minimal activity in cases of recurrent/metastatic HNSCC.^36^ As described above, there is a strong rationale for combining SMAC mimetics with RT and/or immune checkpoint inhibitors. In an exploratory window‐of‐opportunity trial focused on pharmacokinetics and pharmacodynamics of xevinapant, HNSCC patients were treated with xevinapant, cisplatin chemotherapy, or both prior to surgery.^39^ After treatment with xevinapant, tumors demonstrated higher infiltration by CD8+ T cells and higher expression of PD‐1/PD‐L1 and NF‐κB/IAP pathway mediators.^39^ Accordingly, there are multiple ongoing clinical trials combining IAPi with immunotherapy for solid tumors, which may include HNSCC, e.g., the ASTEROID trial with tolinapant + pembrolizumab (NCT05082259) and a trial combining xevinapant with avelumab (NCT03270176).

**TABLE 1:** Inhibitors of apoptosis protein antagonists under clinical study for head and neck squamous cell carcinoma.

Drug name	Route of administration	Phase of study	Mechanism of action
Xevinapant (Debio 1143)	Oral	Phase III	Mimics N‐terminal tetrapeptide of second mitochondria‐derived activator of caspases (SMAC), binds to X‐linked inhibitor of apoptosis (XIAP) and cellular inhibitor of apoptosis 1 and 2 (cIAP1/2)^34^
Tolinapant (ASTX660)	Oral	Phase II	Nonpeptidomimetic small molecule, binds BIR3 domains of XIAP and cIAP1/2^35^
Birinapant (TL32711)	IV	Phase I	Bivalent mimetic of SMAC that binds to cIAP1/2^9^
APG‐1387	IV	Phase I	Bivalent mimetic of SMAC that binds to XIAP and cIAP1/2^24^

**TABLE 2:** Clinical trials of inhibitor of apoptosis protein (IAP) antagonism for head and neck squamous cell carcinoma (HNSCC).

Drug	Phase	Study design	Study population	Registration number^a^	Status	Key findings
Xevinapant	I	Window‐of‐opportunity trial of xevinapant and/or cisplatin chemotherapy prior to surgery	PULA HNSCC patients undergoing surgery	N/A	Completed	PD/PK established. Increased CD8^+^ T cells, PD‐1/PD‐L1, IAP pathway mediator expression
	I/II	Open‐label study with cisplatin chemoradiation (phase I); Randomized, double‐blind, multicenter study, with cisplatin chemoradiation (phase II)	PULA HNSCC, high risk with (+) smoking history	NCT02022098	Completed, published	MTD/RP2D established with CRT. LRC and progress‐free survival higher with xevinapant versus placebo
	III	Randomized, double‐blind, placebo‐controlled multicenter study, with cisplatin chemoradiation	PULA HNSCC, high‐risk human papillomavirus‐negative	NCT04459715	Recruiting	N/A (ongoing, no results posted)
	III	Randomized, double‐blind, placebo‐controlled multicenter study, with adjuvant radiation	PULA HNSCC, cisplatin ineligible	NCT05386550	Recruiting	N/A (ongoing, no results posted)
Tolinapant	Early phase	Open‐label, single‐arm study of tolinapant with definitive or adjuvant radiation therapy (RT)	PULA HNSCC, cisplatin ineligible	NCT05245682	Recruiting	N/A (ongoing, no results posted)
	I/II	Open‐label, dose‐escalation study of tolinapant monotherapy	Lymphoma or advanced solid tumors including HNSCC	NCT02503423	Active, not recruiting	MTD/RP2D established. Minimal activity as monotherapy in HNSCC
Birinapant	I	Open‐label, dose‐escalation study of birinapant monotherapy	Lymphoma or advanced solid tumors including HNSCC	NCT00993239	Completed, published	MTD established
	I	Open‐label, dose‐escalation study of birinapant + re‐RT	Locoregional recurrent HNSCC	NCT03803774	Recruiting	N/A (ongoing, no results posted)
APG‐1387	I/II	Open‐label study of APG‐1387 + toripalimab	Nasopharyngeal carcinoma and other solid tumors	NCT04284488	Recruiting	N/A (ongoing, no results posted)

Abbreviations: MTD, maximum tolerated dose; PULA, previously untreated locally advanced; RP2D, recommended phase 2 dose.

^a^ On ClinicalTrials.gov.

Multiple trials of IAPi + RT are underway or recently completed. Xevinapant was tested as a treatment escalation strategy in addition to high‐dose bolus cisplatin chemotherapy with standard fractionated RT for patients with high‐risk, previously untreated, locally advanced HNSCC. In phase I, the maximum tolerated dose was established.^40^ The follow‐up phase II study was a randomized, placebo‐controlled trial of xevinapant versus placebo in addition to standard cisplatin chemoradiation. Overall survival was not significantly improved, but the study was likely underpowered for that outcome with 48 patients per group. Locoregional control (the primary endpoint) was significantly improved with xevinapant versus placebo.^41^ Remarkably, the two‐year progress‐free survival (PFS) nearly doubled with xevinapant. However, the 2‐year PFS was particularly low for the placebo group at around 40%. A phase III, multicenter, international study (TrilynX study) is currently underway.^34^

IAPi are also under investigation as an alternative radiosensitizer in patients who cannot tolerate standard cisplatin chemotherapy. An early‐phase study underway at our institution combining tolinapant with RT is enrolling cisplatin‐ineligible patients and evaluating safety, survival, and immune activation as primary, secondary, and tertiary outcomes, respectively. Another study using xevinapant in the adjuvant setting for cisplatin‐ineligible patients (XRAY VISION study) is also underway. In the recurrent/metastatic setting, there is one trial open combining birinapant with re‐irradiation (also without chemotherapy) for locally recurrent disease. An upcoming cooperative group trial (NRG HN2201) will compare xevinapant versus cetuximab with definitive RT for cisplatin‐ineligible HNSCC patients.

### 1.6 Future directions and remaining questions

The encouraging activity of IAPi in both HPV‐positive and HPV‐negative diseases, and their unique ability to inhibit the extrinsic cell death pathway suggest that IAPi are a truly novel drug class with great potential for treating HNSCC. Although mutated *TP53* and PD‐L1 expression have been used to guide some therapies, the use of genomic biomarkers for HNSCC is still in its infancy. Based on the poor outcomes—and excellent responses to IAPi—seen in patients with caspase 8 mutations or amplification in *FADD* or *BIRC2/3* genes, this class of drugs offers an opportunity for precision therapy.

HPV‐negative HNSCC warrants treatment escalation in order to improve outcomes. However, IAPi may also be useful as a strategy for de‐escalation for HPV‐positive disease or in situations where more aggressive therapies are not tolerated, such as in the cisplatin‐ineligible population described above. It will take several years and perhaps decades for late‐phase clinical trials to complete accrual and mature. In the meantime, robust preclinical studies will continue to provide mechanistic insights on rational therapeutic combinations that include IAP antagonists.

## AUTHOR CONTRIBUTIONS

**Vikash Kansal:** Conceptualization (equal); writing – original draft (equal); writing – review and editing (equal). **Brendan Logan Clark Kinney:** Conceptualization (equal); writing – original draft (equal); writing – review and editing (equal). **Srijayaprakash Uppada:** Writing – original draft (equal); writing – review and editing (equal). **nabil saba:** Conceptualization (equal); writing – original draft (equal); writing – review and editing (equal). **William Stokes:** Writing – original draft (equal); writing – review and editing (equal). **Zachary Buchwald:** Conceptualization (equal); writing – original draft (equal); writing – review and editing (equal). **Nicole C. Schmitt:** Conceptualization (lead); supervision (equal); writing – original draft (equal); writing – review and editing (equal).

## DISCLOSURE STATEMENTS

Schmitt—*Consulting*: Checkpoint Surgical, Sensorion, Synergy Research.

*Book Royalties*: Plural Publishing.

*Clinical Trial Funding*: Astex Pharmaceuticals.

## Data Availability

N/A.
